# Complete Genome Sequence of *Bifidobacterium kashiwanohense* JCM 15439^T^, Isolated from Feces from a Healthy Japanese Infant

**DOI:** 10.1128/genomeA.00255-15

**Published:** 2015-04-16

**Authors:** Hidetoshi Morita, Hidehiro Toh, Akiyo Nakano, Kenshiro Oshima, Misako Takagi, Wataru Suda, Soichi Tanabe, Masahira Hattori

**Affiliations:** a School of Veterinary Medicine, Azabu University, Sagamihara, Kanagawa, Japan; b Medical Institute of Bioregulation, Kyushu University, Fukuoka, Japan; c Department of Microbiology and Immunology, Teikyo University School of Medicine, Tokyo, Japan; d Graduate School of Frontier Sciences, The University of Tokyo, Kashiwa, Chiba, Japan; e Crossfield-Bio, Inc., Chuo-ku, Tokyo, Japan; f Department of Microbiology and Immunology, School of Medicine, Keio University, Shinjuku-ku, Tokyo, Japan; g Graduate School of Biosphere Science, Hiroshima University, Hiroshima, Japan

## Abstract

We isolated *Bifidobacterium kashiwanohense* JCM 15439 from the feces of a healthy Japanese infant and proposed it as the type strain of a novel species within the genus *Bifidobacterium*. Here, we report the complete genome sequence of this organism.

## GENOME ANNOUNCEMENT

*Bifidobacterium* spp. are Gram-positive, anaerobic, branched rod-shaped bacteria and are frequently isolated from the human intestine. We isolated *Bifidobacterium kashiwanohense* JCM 15439 (= DSM 21854^T^) from the feces of a healthy Japanese infant and proposed it as the type strain of a novel species within the genus *Bifidobacterium* (1). *B. kashiwanohense* is closely related to *Bifidobacterium angulatum*, *Bifidobacterium catenulatum*, *Bifidobacterium dentium*, and *Bifidobacterium pseudocatenulatum* and is thus found to belong to the *Bifidobacterium adolescentis* group (1, 2). The ability of this species to efficiently utilize iron sequestration mechanisms, such as siderophore production and iron internalization, may confer an ecological advantage (3). The basis for enhanced competition against enteropathogens might interestingly be one of the gut infection protective mechanisms.

The genome sequence of JCM 15439^T^ was determined by the whole-genome shotgun strategy using Sanger sequencing (3730xl DNA sequencers) and 454 pyrosequencing (GS-FLX sequencers). We constructed small-insert (2-kb) and large-insert (10-kb) genomic DNA libraries and generated 23,040 (7.7-fold, 3730xl) and 61,480 reads (7.1-fold, GS-FLX) from the *B. kashiwanohense* JCM 15439^T^ genome. The 454 pyrosequencing reads were assembled using the Newbler assembler software. A hybrid assembly of 454 and Sanger reads was performed using the Phred-Phrap-Consed program. Gap closing and resequencing of the low-quality regions were conducted by Sanger sequencing to obtain the high-quality finished sequence. The overall accuracy of the finished sequence was estimated to have an error rate of <1 per 10,000 bases (Phrap score, ≥40). An initial set of predicted protein-coding genes was identified using Glimmer 3.0 (4). Genes consisting of <120 bp and those containing overlaps were eliminated. The tRNA genes were predicted by tRNAscan-SE (5), and the rRNA genes were detected by BLASTn search using known *Bifidobacterium* rRNA sequences as queries.

The genome sequence of *B. kashiwanohense* JCM 15439^T^ consists of a circular chromosome of 2,337,234 bp and two cryptic plasmids (pBBKW-1 and pBBKW-2). As previously reported (6), pBBKW-1 (7,716 bp) is a cointegrate plasmid, and pBBKW-2 (2,920 bp) is a theta-type replicating plasmid. The chromosome contains 1,945 predicted protein-coding genes. We compared the genome of JCM 15439^T^ with that of *B. kashiwanohense* PV20-2 (2,370,978 bp), which was isolated from the feces of an anemic Kenyan infant (7). A genome alignment showed a high level of sequence similarity between the two strains. Of the 1,945 protein-coding genes, 1,476 (76%) were shared by the two strains. The remaining 469 protein-coding genes were dominated by hypothetical proteins or proteins of unknown function but contained several carbohydrate utilization gene clusters (carbohydrate transporter, glycosyl hydrolase, and transcriptional regulator). On the other hand, PV20-2 contained a clustered regularly interspaced short palindromic repeat (CRISPR) region and the urease gene cluster (AH68_03530 to AH68_03580), which were not found in JCM 15439^T^. The urease gene cluster was also found in the genome of *Bifidobacterium longum* subsp. *infantis* (8). The genome information of this recently isolated species will be useful for further studies of its physiology, taxonomy, and ecology.

### Nucleotide sequence accession numbers. 

The sequence data for the genome have been deposited in DDBJ/GenBank/EMBL under the accession numbers AP012327 (chromosome), AB713428 (pBBKW-1), and AB713429 (pBBKW-2).
